# The impacts of high-fidelity and virtual reality simulation on the development of non-technical skills in healthcare students and professionals: protocol for a systematic review

**DOI:** 10.1136/bmjopen-2025-115169

**Published:** 2026-05-13

**Authors:** William Hunt, Selina Lock, Adam Bonfield, Jeremy Howick

**Affiliations:** 1 Stoneygate Centre for Empathic Healthcare, Leicester Medical School, University of Leicester, Leicester, UK; 2 Library and Learning Services, University of Leicester, Leicester, UK; 3 Honorary Lecturer in Simulation, Leicester Medical School, University of Leicester College of Life Sciences, Leicester, England, UK

**Keywords:** Virtual Reality, MEDICAL EDUCATION & TRAINING, Health Education, Health Workforce

## Abstract

**Abstract:**

**Introduction:**

Deficiencies in non-technical skills—including communication and leadership—are well-established causes of clinical errors in healthcare. Healthcare students and professionals increasingly use high-fidelity and virtual reality (VR) simulation to replicate clinical practice, through immersive and realistic scenarios in a risk-free teaching setting. However, there is no up-to-date, high-quality synthesis of the effects of high-fidelity and VR simulation on non-technical skills for healthcare students and professionals. A systematic review and meta-analysis of this literature is required to enhance the current knowledge.

**Methods:**

This protocol has been reported according to the Preferred Reporting Items for Systematic Review and Meta-Analysis Protocols (PRISMA-P) 2015 statement. We will include randomised trials and other controlled studies that report differences in non-technical skills between high-fidelity and VR simulation. We will search MEDLINE, Scopus, EMBASE, ERIC and CINAHL, from database inception. We will also search reference lists and contact experts to identify additional studies. Two independent reviewers will screen titles and abstracts, review full texts, and extract data. Discrepancies will be resolved through discussion, with a third reviewer if necessary. For randomised trials, we will use the Cochrane Risk-of-Bias 2.0 (RoB2) tool to evaluate the risk of bias in the included studies. For non-randomised studies, we will use the Risk Of Bias In Non-randomized Studies (ROBINS-1) assessment tool. If appropriate, meta-analysis will be performed. We will analyse continuous outcomes using weighted mean differences (with 95% CIs) or standardised mean differences (with 95% CIs) if different measurement scales are used. We will use subgroup and sensitivity analyses to explore heterogeneity. The overall certainty of evidence will be assessed using the GRADE (Grading of Recommendations, Assessment, Development and Evaluations) tool.

**Ethics and dissemination:**

Ethical approval is not applicable for this study because no primary data have been collected. This review will be disseminated through peer-reviewed publication and presented at conferences to inform ongoing educational practises.

**PROSPERO registration number:**

CRD420251136479.

**STRENGTHS AND LIMITATIONS OF THIS STUDY**

- The most comprehensive and up-to-date systematic review investigating the impacts of high-fidelity and virtual reality (VR) simulation on non-technical skills across a wide range of healthcare students and professionals.
- The first systematic review to perform direct comparison between high-fidelity and VR simulation and their effects on non-technical skills.
- Proposes a rigorous methodology, reported using the preferred reporting items for systematic review and meta-analysis (PRISMA) statement.
- We will use a broad search strategy, as aided by an experienced librarian, using multiple high-quality databases to ensure all relevant data are included.
- A potential limitation is that the evidence provided by this systematic review may be affected by the number or quality of studies included.

## Introduction

 Non-technical skills are a set of skills, exhibited by both individuals and teams, which are needed to reduce errors and improve human performance within complex systems.^1^ These skills include cognitive components such as decision-making, problem solving, critical thinking, clinical judgement and situation awareness, as well as social components such as teamwork, communication and leadership.^2^ Healthcare educators must foster these abilities throughout training to develop safe and effective professionals.^3^

Unfortunately, evidence shows that failures in non-technical skills are a leading cause of clinical errors in healthcare. Poor communication alone causes as many as one in 10 patient safety incidents.^4 5^ This can result in significant costs to healthcare systems and adversely affects patient outcomes.^6^ Educators must implement interventions directed at teaching such non-technical skills to safeguard patient safety.^6^,^8^ Furthermore, regulatory and educational bodies increasingly recognise the need for such educational interventions.^9 10^

Despite a variety of educational interventions—ranging from traditional lectures and role-playing to simulation, and more recently virtual reality (VR) and artificial intelligence (AI) based tools—errors arising from poor non-technical skills remain common.^11^,^13^ Evaluating the effectiveness of these training methods is therefore a key priority.^14^

Simulation provides a realistic and controlled environment to practise non-technical skills without compromising patient safety.^15 16^ Widely applied across healthcare, simulation supports both cognitive and psychomotor development.^17^ Evidence suggests that simulation both enhances the quality of patient care and improves outcomes.^18 19^

Simulation varies in levels of fidelity.^20^ Low-fidelity simulation typically refers to task trainers or static models, such as a manikin arm for practising venepuncture. Comparatively high-fidelity simulation involves advanced technology, such as interactive patient simulators, that can generate a wide variety of physiological responses to stimuli or interventions.^21 22^ This enhances interactivity between learners and educators, and provides realistic scenarios where non-technical skills can be developed.^21^

VR expands the scope of high-fidelity simulation by offering an alternative method for the delivery of interactive simulation scenarios.^23^ VR simulation refers to either screen-based applications or immersive virtual environments (eg, wearing a head-mounted display) through which learners can participate in scenarios.^24^ With rapid progress in AI, the use of VR simulation is increasingly being integrated into medical education.^25^

AI has enabled the development of engaging learning environments and enhances simulation by providing adaptive, personalised learning experiences and real-time feedback.^26^ There is also potential for AI-based systems to analyse real-time learners’ performance data, adjusting scenarios, ensuring learners are challenged in weaker areas.^27^ In combination with VR, AI has the potential to create highly immersive and individualised training experiences which avoid repetition.^28^ These emerging technologies offer promising avenues for developing non-technical skills. However, evidence regarding their effectiveness remains limited and a synthesis is therefore warranted.^29 30^

Three previous systematic reviews address the impact of high-fidelity or VR simulation on non-technical skills. However, no systematic review has directly compared the effect of the two educational modalities. Lewis *et al* and Vangone *et al* suggest that high-fidelity simulation can improve non-technical skills compared with alternative teaching methods but highlight limited and heterogeneous evidence.^31 32^ Bracq *et al* performed a qualitative synthesis assessing the role of VR simulation in non-technical skills training across healthcare professionals and identified few studies that measured its impact.^30^ However, this review did not include quantitative data about the effects of high-fidelity simulation, leaving the effects of the different methods uncertain.

These evidence gaps create uncertainty about whether high-fidelity simulation represents the most effective approach to develop non-technical skills, particularly as emerging technologies such as AI and VR expand the possibilities for simulation-based education.^33^ A comprehensive and updated synthesis of these studies, including comparison between students and healthcare professionals, is necessary to understand the roles of high-fidelity and VR simulation in developing non-technical skills, both in the earlier stages of healthcare professional training and during continuing professional development.

### Aim

To compare the effectiveness of VR and high-fidelity simulation for improving the non-technical skills of healthcare students and professionals.

## Methods

This protocol has been reported according to the Preferred Reporting Items for Systematic Review and Meta-Analysis Protocols (PRISMA-P) 2015 statement.^34^

### Eligibility criteria

The selection process of the studies will be based on the Population, Intervention, Comparison, Outcome (PICO) framework, see table 1.

**Table 1:** Inclusion and exclusion criteria using PICO and other relevant criteria

Element	Inclusion	Exclusion
Population	Healthcare professionals and students	–
Intervention	Virtual reality simulation	–
Comparison	High fidelity simulation	–
Outcomes	Non-technical skills including teamwork, clinical judgement, critical thinking, leadership, communication, situation awareness, decision-making and problem solving	–
Study design	Randomised controlled trials Non-randomised controlled studies	Observational studies Systematic reviews Narrative reviews
Language	Any	–

PICO, Population, Intervention, Comparison, Outcome.

### Participants

Any healthcare professionals or healthcare students (undergraduate or postgraduate).

### Intervention

The research intervention is VR simulation.

### Control

The control is high-fidelity simulation.

### Outcome

The quantitative assessment, using validated scoring systems, of any non-technical skills will be included as outcomes. These include decision-making, problem solving, clinical judgement, critical thinking, situation awareness, teamwork, communication and leadership.

### Study design

Only randomised and non-randomised controlled studies that compare non-technical skills between VR and high-fidelity simulation will be considered for review. Uncontrolled observational studies will not be included. No language or date limits will be imposed on the search. There will be no restriction by type of setting.

### Language

We will include articles reported in any language.

### Information sources

Literature search strategies will be developed using medical subject headings (MeSH) and text words relating to our systematically constructed based ‘Population, Intervention, Comparison, Outcome’ framework (PICO). We will search MEDLINE, EMBASE, Scopus, ERIC and CINAHL. We will search these databases from inception up to 1 December 2025. We will also search reference lists of included studies and contact experts to identify additional studies.

### Search strategy

The specific search strategies will be created by a health sciences librarian with expertise in systematic review searching. The MEDLINE strategy will be developed with input from the project team. A draft MEDLINE search strategy is included in online supplemental appendix 1. After the MEDLINE strategy is finalised, it will be adapted to the syntax and subject headings of the other databases. PROSPERO will be searched for ongoing or recently completed systematic reviews.

### Study records: selection process

Two review authors will independently screen the titles and abstracts. We will obtain full reports for all titles that appear to meet the inclusion criteria or where there is any uncertainty. Review author pairs will then screen the full text reports and decide whether these meet the inclusion criteria. Any discrepancies will be resolved by discussion with the presence of a third author if necessary. We will seek additional information from study authors where necessary to resolve questions about eligibility. We will record the reasons for excluding trials. Review authors will not be blinded to journal titles, study authors or institutions.

### Study records: data management

Literature search results will be exported to Rayyan, an internet-based software programme that facilitates collaboration between reviewers during the study selection process.^35^ The platform will be used for citation importing and screening, full text review, study selection and quality assessment. Prior to the formal screening process, a calibration exercise will be undertaken to pilot and refine the screening questions. We will provide training to new members of the review team not familiar with the Rayyan software and the content area prior to the start of the review.

### Study records: data collection process

Data extraction will take place using a bespoke extraction sheet tailored to individual study design using Microsoft Excel. Two independent reviewers will extract the study data. We will conduct a calibration exercise before starting the review. This will involve both reviewers independently extracting five studies and ensuring consistency across reviewers. During extraction, discrepancies will be resolved by discussion with an independent adjudicator helping to resolve any disagreements. We will contact study authors to resolve any uncertainties.

### Data items: general

We will collect data on:

- The report: author, year and source of publication.
- Study characteristics: aim, duration of participation, units of allocation, ethical approval, sample size.
- Participant demographic data: healthcare professional background, level of training, student status, study setting, general demographics, inclusion criteria, exclusion criteria, method of recruitment of participants, informed consent obtained, total number randomised (if applicable), withdrawals.
- Methodology: study design, treatment assignment mechanism, interventions including description and delivery, high fidelity simulator used, VR model used, and statistical methods.
- Outcome data including types of non-technical skills assessed, tools for assessment, time points for assessment and subgroups measured.

### Data items: outcomes and prioritisation

The primary outcome of interest is the effect of VR Simulation versus high-fidelity simulation on any non-technical skills. Any randomised controlled trial or non-randomised controlled study that meets the inclusion criteria and uses validated quantitative assessment of non-technical skills will be included. We will include outcomes assessing individual non-technical skills, including decision-making, clinical judgement, critical thinking, problem solving, situation awareness, teamwork, communication and leadership. We expect non-technical skills assessments to occur in the form of validated assessment tools, performance checklists or self-reporting scales. Though several validated measures do exist, we expect a degree of heterogeneity in the outcome tool or instruments used to measure non-technical skills.^36^,^39^ The outcome prioritisation plan may be expanded based on the scope of the literature that is identified.

### Risk of bias assessment

For randomised controlled trials, we will use the Cochrane Risk-of-Bias 2.0 (RoB2) tool to evaluate the risk of bias in the included studies.^40^ For non-randomised trials, we will use the Risk Of Bias In Non-randomized Studies (ROBINS-1) assessment tool.^41^ Two reviewers will assess the risk of bias after the initial data screening and prior to data extraction. The content of the study will inform judgements regarding the risk of bias for each of the questions outlined on the checklist. Two reviewers will perform this assessment independently. Where there is any discrepancy, an independent third reviewer will help resolve discrepancies. For randomised trials, graphical representation of potential bias will be generated using Robvis, an online tool designed to help visualise the risk-of-bias assessments for systematic reviews.^42^

### Synthesis methods

If studies are sufficiently homogenous, we will conduct meta-analyses. If so, data will be pooled with summary estimates and 95% CIs. A random effects model will be used to account for the likely diversity in individual study populations and interventions, as well as differing controls between studies. Randomised controlled trials or non-randomised controlled studies with continuous outcomes will be analysed using weighted mean differences with 95% CI, or standardised mean differences with 95% CI. For dichotomous outcomes, we will analyse using ORs. Where data are not sufficient or suitable for meta-analysis, a systematic narrative summary will be provided. Data from individual studies will be presented in a summary table detailing study design, population, intervention, control and results. Forest plots will be used to visually display the results of individual studies and meta-analyses. Between-study heterogeneity will be assessed using the visual inspection of forest plots and will be quantified using the I^2^ statistic as recommended by the Cochrane Handbook for systematic reviews of interventions.

### Subgroup and sensitivity analyses

If feasible, with sufficient data and study homogeneity, we will aim to conduct subgroup analyses via meta-analysis. These proposed subgroups will be:

- Age (under 30 vs over 30).
- Sex.
- Sociodemographic variables.
- Study design types (randomised trials vs non-randomised controlled studies).
- Different healthcare professional groups (eg, medical, nursing, physiotherapy, paramedic, midwife or operating department practitioner (ODP) students and professionals).
- Students and professionals.

Further subgroup analysis will be guided by the data collected in the extraction process. These subgroup analyses will help explore the wider impacts of high-fidelity and VR simulation on non-technical skills across the range of healthcare professional and student groups.

We will perform sensitivity analyses according to overall study quality to assess the robustness of findings, with studies identified as high risk of bias being omitted. The impact of assumed or imputed data will also be analysed in a sensitivity analysis where studies in which data were imputed are removed.

### Publication bias assessment

The presence of publication bias will be evaluated by the visual inspection of funnel plots. Any asymmetry within these plots may indicate potential publication bias.

### Certainty assessment

The overall certainty of evidence will be assessed using the GRADE (Grading of Recommendations, Assessment, Development and Evaluations) tool.^43^

### Ethics and dissemination

Ethical approval is not required, as these are secondary analyses and data are used in aggregated, de-identified form.

This review will be disseminated through peer-reviewed publication and presented at conferences to inform ongoing educational practises.

## Supplementary material

10.1136/bmjopen-2025-115169 online supplemental file 1
