# Supplementary material for: The impacts of high-fidelity and virtual reality simulation on the development of non-technical skills in healthcare students and professionals: protocol for a systematic review
Source: BMJ Open. 2026 May 13;16(5):e115169. doi: 10.1136/bmjopen-2025-115169 (PMC13182308; doi:10.1136/bmjopen-2025-115169)
Supplement: online supplemental file 1 [file bmjopen-16-5-s001.docx]

# Appendix 1: PIcO Search adapted for Medline

| PIcO | Number | Search Term |
| --- | --- | --- |
| Population | 1 | ((health or healthcare or "health care") adj (professional* or personnel or practitioner* or staff or provider or student*)).ti,ab,kf. |
|  | 2 | (physician* or clinician* or nurs* or doctor* or medical student* or physiotherapist* or "physical therapist*" or paramedic* or EMT* or "emergency medical technician*" or anaesthetist* or anasthetist* or anesthesiologist* or surgeon* or "operating department practi*" or ODP or midwif* or midwives or "patient care team" or trainee* or resident* or registrar* or intern* or fellow*).ti,ab,kf. |
|  | 3 | Health Personnel/ or Students, Medical/ or Students, Nursing/ or exp Nurses/ or Physicians/ or Physical Therapists/ or Paramedics/ or Anesthetists/ or Surgeons/ or Operating Room Nursing/ or Midwifery/ or Patient Care Team/ or "Internship and Residency"/ or Education, Medical, Graduate/ |
|  | 4 | 1 or 2 or 3 |
| Intervention | 5 | High Fidelity Simulation Training/ or Simulation Training/ |
|  | 6 | ((("high fidelity" or "high-fidelity") adj2 simulat*) or simulator fidelity*).ti,ab,kf. |
|  | 7 | Virtual Reality/ or Smart Glasses/ or Computer Simulation/ |
|  | 8 | (virtual adj (realit* or environment*)).ti,ab,kf. |
|  | 9 | ("augmented reality" or "mixed reality" or "extended reality" or AR or MR or XR or "serious game*" or "serious simul*" or "head mounted display" or HMD or "head-mounted display*").ti,ab,kf. |
|  | 10 | 5 or 6 or 7 or 8 or 9 |
| Outcome | 11 | ("team work*" or "critical thinking" or "thinking critical*" or "clinical judgement*" or "clinical judgment" or "situational awareness" or "situation Awareness" or "decision making" or decision-making or "leadership" or "communication" or "non technical skill*" or "nontechnical skill*" or "non-technical skill*" or crewresource* or "crew resource* management" or CRM).ti,ab,kf. |
|  | 12 | ((time or task) adj1 manag*).ti,ab,kf. |
|  | 13 | Clinical Decision-Making/ or Decision Making/ or Awareness/ or Leadership/ or Communication/ or Time Management/ or Thinking/ or Judgment/ |
|  | 14 | 11 or 12 or 13 |
|  | 15 | 4 and 10 and 14 |
