# Supplementary material for: The impacts of high-fidelity and virtual reality simulation on the development of non-technical skills in healthcare students and professionals: protocol for a systematic review
Source: BMJ Open. 2026 May 13;16(5):e115169. doi: 10.1136/bmjopen-2025-115169 (PMC13182308; doi:10.1136/bmjopen-2025-115169)
Supplement: Supplementary file 2 [file bmjopen-2025-115169.reviewer_comments.pdf]

PEER REVIEW HISTORY

BMJ Open publishes all reviews undertaken for accepted manuscripts. Reviewers are asked to complete a checklist review form and are provided with free text boxes to elaborate on their assessment. These free text comments are reproduced below.

ARTICLE DETAILS

Title (Provisional)

The impacts of high-fidelity and virtual reality simulation on the development of non-technical skills in healthcare students and professionals: Protocol for a systematic review

Authors

Hunt, William; Lock, Selina; Bonfield, Adam; Howick, Jeremy

VERSION 1 - REVIEW

Reviewer **1**

Name **Yang, Ting**

Affiliation **Affiliated Hospital of Nanjing University of Chinese Medicine**

Date **02-Feb-2026**

COI **None**

Dear Editor

Thank you very much for giving me this valuable opportunity to review this study. This study provides a good introduction to the systematic evaluation of VR and high-fidelity simulation in clinical education, which is quite aligned with our team's research direction. Therefore, we highly respect the editor's professionalism and rigor.

We would also like to thank the researchers for this article. We believe that you must have conducted extensive literature searches and clinical reflections to arrive at this topic selection. The novelty of the topic, the standardized writing, and the rigorous methodology have greatly inspired us.

There is a minor issue: the paragraph formatting in the article is "left-aligned" rather than "justified". We believe that adjusting this minor issue can meet the publication standards.

I am a Chinese scholar. Your Prime Minister has just visited China, further strengthening the relationship between our two countries. Welcome to visit China and give lectures.

Reviewer **2**

**Name**	**Assis Neves Dantas, Rodrigo**
**Affiliation**	**Federal University of Rio Grande do Norte, Nursing**
**Date**	**21-Feb-2026**
**COI**	**None**

Initially, I would like to congratulate the authors on the excellent work presented. The protocol addresses a relevant and current topic, exploring a significant gap in the literature, with potential scientific and clinical impact, especially for professionals and students in the health field. The rationale is well-constructed and clearly demonstrates the need for the proposed review.

Regarding the study method, the manuscript is solid and aligned with international guidelines for systematic review protocols. The objective is clear, the eligibility criteria are consistent with the research question, and the search strategy is adequate and reproducible. The selection process, data extraction, and risk of bias assessment demonstrate rigor and transparency. Furthermore, the data synthesis plan, including the provision for meta-analysis when pertinent and the assessment of the certainty of the evidence, further strengthens the protocol and demonstrates a commitment to the methodological quality of the work.

Therefore, I consider the protocol to be methodologically adequate, well-structured, and possessing sufficient scientific merit for publication.

VERSION 1 - AUTHOR RESPONSE

Reviewer 1 – Dr. Ting Yang

Comment 1: There is a minor issue: the paragraph formatting in the article is "left-aligned" rather than "justified". We believe that adjusting this minor issue can meet the publication standards.

Many thanks for your comments and suggestions. We have ensured that the paragraph formatting has been updated to ‘justified’ to meet the publication standards.

Reviewer 2 - Dr. Rodrigo Assis Neves Dantas

Many thanks for your rigorous review and kind comments. We did not find anything actionable in these comments.
